# Identification of *AGO2* and *PLEC* genes polymorphisms in Hu sheep and their relationship with body size traits

**DOI:** 10.1080/10495398.2023.2295926

**Published:** 2023-12-27

**Authors:** Jia Liu, Wenxin Zheng, Weimin Wang, Xiaobin Yang, Yongliang Huang, Panpan Cui, Zongwu Ma, Xiwen Zeng, Rui Zhai, Xiuxiu Weng, Weiwei Wu, Xiaoxue Zhang

**Affiliations:** a College of Animal Science and Technology, Gansu Agricultural University, Lanzhou, China; b Institute of Animal Husbandry Quality Standards, Xinjiang Academy of Animal Sciences, Urumqi, Xinjiang, China; c The State Key Laboratory of Grassland Agro-Ecosystems, College of Pastoral Agriculture Science and Technology, Lanzhou University, Lanzhou, China; d Institute of Animal Science, Xinjiang Academy of Animal Sciences, Urumqi, Xinjiang, China

**Keywords:** Hu sheep, *AGO2*, *pLEC*, body size traits, qRT-PCR

## Abstract

The body size traits are major traits in livestock, which intuitively displays the development of the animal’s bones and muscles. This study used PCR amplification, Sanger sequencing, KASPar genotyping, and quantitative real-time reverse transcription PCR (qRT-PCR) to analyze the Single-nucleotide polymorphism and expression characteristics of Argonaute RISC catalytic component 2 (*AGO2*) and Plectin (*PLEC*) genes in Hu sheep. Two intron mutations were found in Hu sheep, which were *AGO2* g.51700 A > C and *PLEC* g.23157 C > T, respectively. Through association analysis of two mutation sites and body size traits, it was found that *AGO2* g.51700 A > C mainly affects the chest and cannon circumference of Hu sheep of while *PLEC* g.23157 C mainly affects body height and body length. The combined genotypes of *AGO2* and *PLEC* genes with body size traits showed SNPs at the *AGO2* g.51700 A > C and *PLEC* g.23157 C > T loci significantly improved the body size traits of Hu sheep. In addition, the *AGO2* gene has the highest expression levels in the heart, rumen, and tail fat, and the *PLEC* gene is highly expressed in the heart. These two loci can provide new research ideas for improving the body size traits of Hu sheep.

## Introduction

Sheep (*Ovis aries*) are important livestock species. Since the Neolithic age, it has undertaken the responsibility of providing meat, fur and milk for humans^1^. Hu sheep is a unique lamb breed in China, mainly distributed in the Jiaxing and Taihu areas of Zhejiang Province, with the advantage of early body development^2^. The body size traits of sheep are controlled by micro effect polygenes. The selection of gene loci that can improve the body size traits of sheep is of great significance to improve the economic benefits of sheep breeding.

AGO2 is the only member with catalytic activity in the Argonaute family, it is the main component of the RNA-induced silencing complex. The human AGO family includes four different proteins: AGO1, AGO2, AGO3, and AGO4^3^. This family gene is not only widely distributed in animals and plants^4–8^, but also can change the structure of the 5′ terminal of some viruses^7^. Although they have strong homology, it has been proved that only *AGO2* has the effect of post-translational modification. It can not only maintain miRNA homeostasis^8^ but also change miRNA activity to a certain extent^9^. The human *AGO2* gene and the miRNAs associated with it are able to regulate gene expression in almost all immune cells, with regulatory effects on both specific and nonspecific immunity^10^, and the *AGO2* gene is overexpressed in various cancers^11^. The regulatory effects of the *AGO2* gene on normal growth and obesity are different. Under normal physiological conditions, *AGO2* in the liver can promote the functions of energy metabolism, lipid metabolism, and glucose metabolism in mice, which further affects the objectivity and fat deposition of mice (Zhang et al.)^12^. In addition, hepatic *AGO2* regulates peroxisome proliferator-activated receptor α (PPAR α). In severe obesity, liver-specific *AGO2* efficacy in mice exhibits an increased expression of target genes regulating liver oxidative metabolism, thereby reducing obesity to a certain extent^13^.

*PLEC* is a member of the plakin protein family, encoding plectin (PLEC). PLEC is a bifunctional histidine kinase. PLEC has binding sites for all types of mediating intermediate filament (IF) subunit proteins and plays an important role in connecting and anchoring the organization and performance of cells^14^. Thus the main function of PLEC in the animal body is to connect the individual components of the cytoskeleton. The *PLEC* gene is widely distributed in animal body, mainly exists in skeletal muscle and plays its physiological function^15–18^. It is reported that its missense, frame-shift, and splice site mutations will cause nearly 100 disease-causing of human diseases^15^. In addition, some studies have shown that *PLEC* can promote muscle cell differentiation and proliferation, inhibit its apoptosis, and regulate skeletal muscle development^19–21^.

Marker-assisted selection (MAS) can be used to improve sheep body size traits and shorten breeding time. MAS is a way to directly identify target genes, which can directly identify genes that control traits^22^. MAS has a wide range of applications in agriculture. MAS is an effective means to quickly improve the yield, product quality, stress resistance, and disease resistance of various crops, such as wheat, cotton, rice, etc^23–28^. It is also one of the ways to quickly and accurately improve the production, reproductive performance and reduce adverse traits of poultry and livestock such as chickens, cows and sheep^29–32^. Among them, SNP (Single Nucleotide Polymorphisms) is the most commonly used DNA sequence based molecular marker method for improving livestock traits^33–36^. SNP is not only the most common form of genetic variation in nature but a more in-depth aspect of genetics. Missense mutation can change the amino acid types encoded, but the frequency is extremely low. The vast majority of mutations are silent mutations. Silent mutations do not alter the type of amino acids and do not affect protein function. However, silent mutations can affect protein levels or conformation by modifying mRNA stability, miRNA binding sites, translation efficiency, or splicing regulatory sites, thereby affecting phenotype^37^. Therefore, even though it is generally believed that the change of protein-coding genes is the cause of the differences in animal phenotypes, silent mutations also have an undeniable role in regulating animal growth traits.

The *AGO2* gene, as a candidate gene that affects the body size of cattle, shows an increasing trend in carcass weight of cattle^38^. In previous studies, it was found that the *PLEC* gene was differentially expressed in the muscle samples of Ningxiang Pigs in common for day 1 vs. day 60 and day 60 vs. day 210. This suggests that the *PLEC* gene may serve as a candidate gene affecting muscle development^39^. Carcass weight and muscle development are both growth traits of domestic animals, so these two genes were selected to investigate the impact of these two genes on Hu sheep. The purpose of this study was to find the Single-nucleotide polymorphism sites of *AGO2* and *PLEC* genes, and to explore the effect of two SNPs on the body size traits of Hu sheep. In addition, the expression levels of two genes in different tissues were analyzed to verify the tissues in which they mainly play a role. In order to further investigate the expression differences among different genotypes, the expression levels of individuals with different genotypes in the heart and muscles were tested. Therefore, this study can provide a theoretical basis for molecular breeding and body size research of Hu sheep.

## Materials and methods

### Statement of ethics

The animal study was reviewed and approved by the Animal Care and Use Committee of the Gansu Agricultural University. License No. 2012-2-159.

### Experimental animals and DNA extraction

1318 male Hu sheep of 56-day old were weaned and vaccinated and then transported to Minqin Defu Agriculture Co. Ltd. for centralized feeding, free feeding and drinking, and unified management. After the domestication period (14 d) and the pre-experiment period (10 d), the 100-day experiment was started. The feed in the experiment is the same brand of pellet feed. On the morning of the 80th, 100th, 120th, 140th, 160th and 180th day, the body size traits (body height, body length, chest circumference and cannon circumference) of all experimental sheep were measured and recorded. 5 mL blood was collected from the jugular vein of each sheep, and the blood DNA was extracted using an Easy Pure blood genetic DNA kit (TransGen Biotech, Beijing, China) and stored according to the instructions^37^.

### SNP identification and genotyping

Referring to the sequence of *AGO2* and *PLEC* genes (GenBank: NC_040260.1 and NC_056062.1), primers were designed using Primer 5. Table 1 showed the sequence and optimal annealing temperature of *AGO2* and *PLEC* genes. The SNP sites of these two genes were identified by amplification and sequencing of 10 randomly mixed sheep DNA samples. DNA was extracted using the Beijing TransGen Biotechnology Kit was dissolved in an elution buffer. A total of 35 μL PCR amplification systems (17.5 μL Mix, 14 μL ddH_2_O, 1.05 μL Forward and Reverse primers, 1.4 μL genomic DNA) for genetic thermal gradient PCR. PCR amplification parameters refer to previous studies^37^. The *AGO2* and *PLEC* genes were genotyped by using competitive allele specific FRET-based PCR (KASPar). 1297 *AGO2* and 1313 *PLEC* individuals were genotyped, respectively. 1276 individuals were successfully genotyped with both *AGO2* and *PLEC* genes.

**Table 1.** Forward and reverse primers, optimal annealing temperature and product length for the *AGO2* and *PLEC* genes.

Gene	Primer Name	Primer sequence (5′–3′)	Tm(°C)	Size(bp)
AGO2	AGO2-F	CTGCCCTGTCACCTGCAAG	61	926
	AGO2-R	GAGACACCCCAGACCCGGAA		
PLEC	PLEC-F	GCTGCATTCAGTCCCTAGTCA	56	880
	PLEC-R	CTAATCGTACGGTAACCTGCT		

### Tissue expression analysis

The heart, liver, spleen, lung, kidney, rumen, duodenum, muscle, lymph and tail fat tissue samples of six experimental Hu sheep were collected to identify the expression of *AGO2* and *PLEC* genes in different tissues of experimental animals. The total RNA in the tissue samples were extracted by TransZol (TransGen Biotech, Beijing, China) and then transcribed into cDNA by using a reverse transcriptase kit (Takara, Dalian, China). According to the existing research, the experimental reagents were mixed and the parameters were set for qRT-PCR^37^. The 2−^ΔΔCt^ method was used to analyze the data^40^.

### Statistical analysis

The following models in SPSS 23.0 were used to analyze the association between the genotype and body size traits of experimental Hu sheep:

$$Y_{\text{ijkl}}=\mu +G_{i}+B_{j}+S_{k}+C_{l}+\varepsilon_{\text{ijkl}},$$

$$Y_{\text{imjkln}}=\mu +G_{i}+G_{m}+F_{j}+S_{k}+C_{l}+C_{n}+\varepsilon_{\text{imjkln}},$$

where Y*_ijkl_* and Y*_imjkln_* was the phenotypic observation value of body size traits, μ is the mean, G*_i_* represents the influence of genotypes (*i*=AA, AC or CC and *i*=TT, TC or CC), B*_j_* represents the influence of batchs (*j* = 2, 3, ……, 8); S*_k_* represents the influence of seasons (*k* = 1, 2); C*_l_* refers to the effect of combination, ε*_ijkl_* and ε*_imjkln_* was the residual corresponding to the observed value of the traits, G*_i_*, B*_j_* and S*_k_* were fixed effects. Significant differences were determined using the LSD-t test. When *p* < .05, the difference is considered significant, that is, genotype has a significant impact on phenotype^37^.

## Results

### SNP scanning of Hu sheep AGO2 and PLEC genes

**Figure 1.**
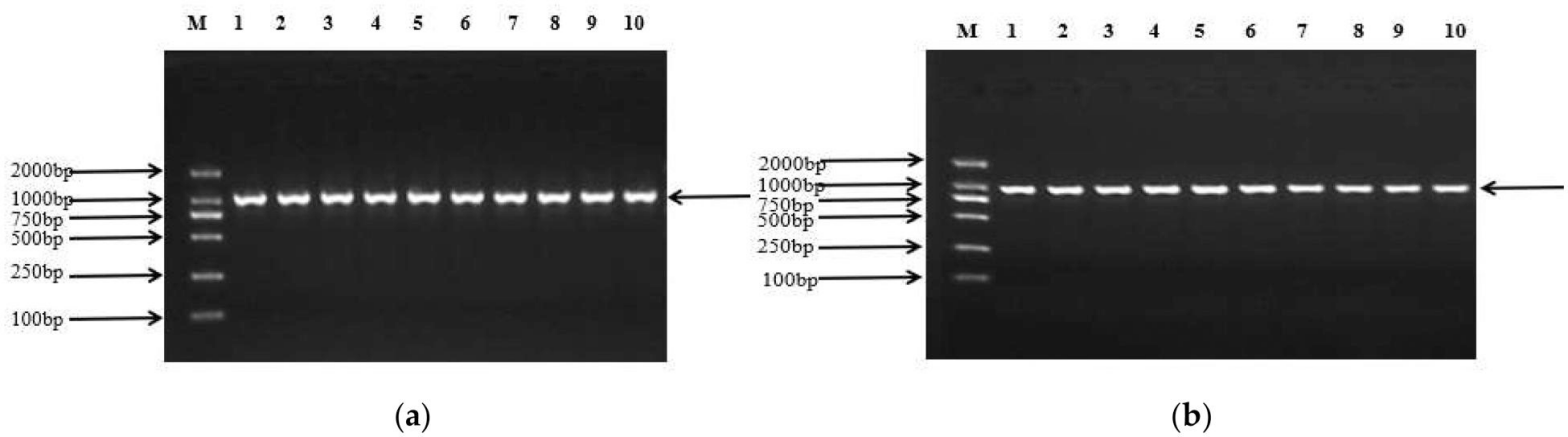
PCR amplification of *AGO2* (a) *and PLEC* (b). Lanes 1–10: target fragments; M: DL2000 DNA Marker.

**Figure 2.**
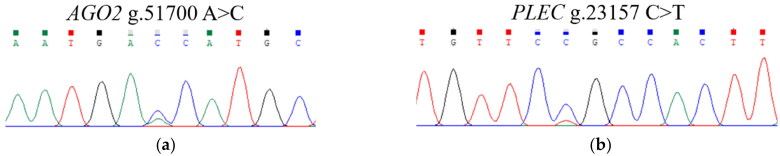
Sequencing peak map of Hu sheep *AGO2* (a) and *PLEC* (b) genes loci.

**Figure 3.**
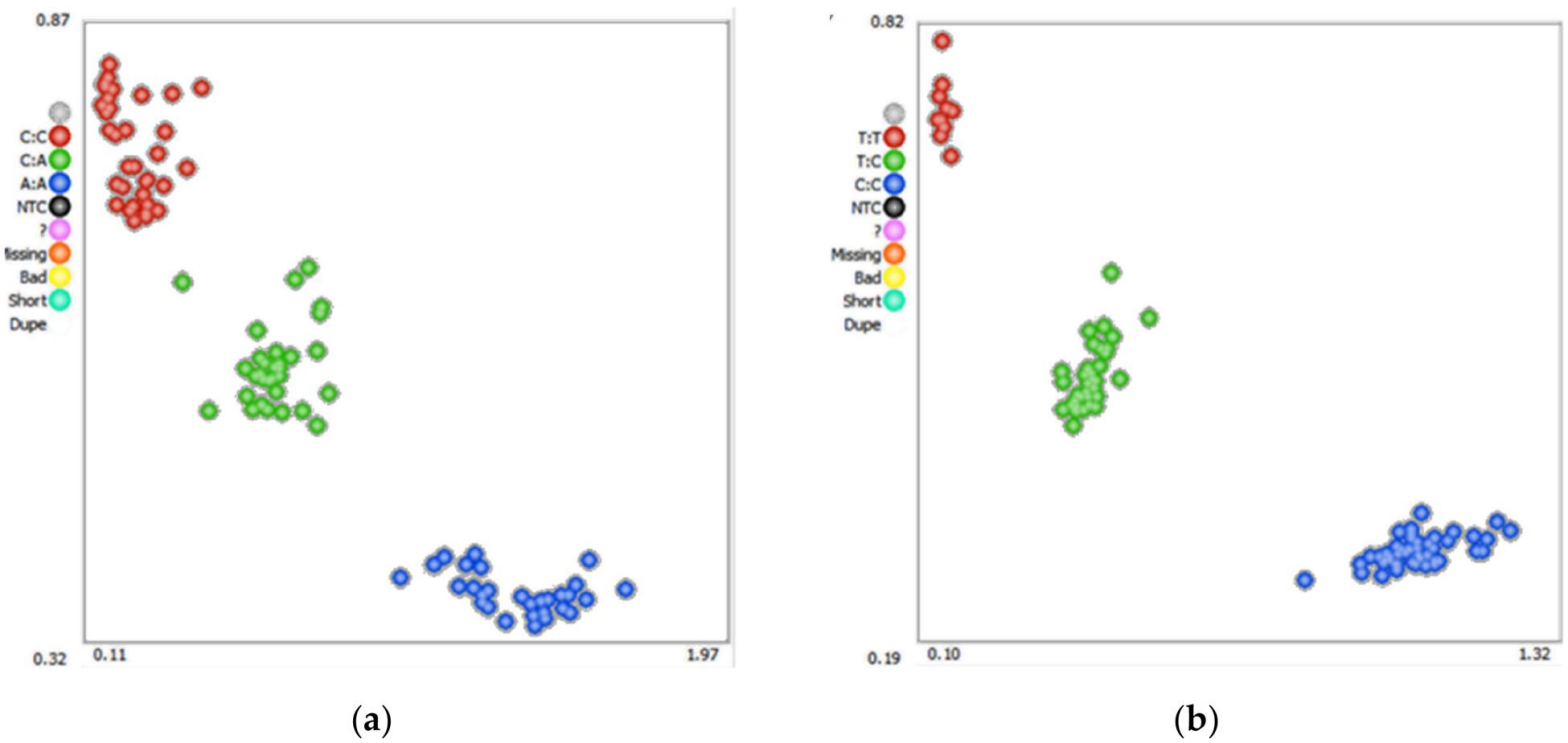
Genotyping of sheep *AGO2* g.51700 a > C (a) and *PLEC* g.23157 C > T (b) SNPs using Kaspar technology.

Based on the primers we’ve designed, 926 bp and 880 bp sequences of *AGO2* and *PLEC* genes were amplified from the mixed DNA of 10 randomly selected experimental animals, respectively (Fig. 1). KASPar genotyping was used to identify one mutation site in each of the two genes, namely *AGO2* g.51700 C > A and *PLEC* g.23157 T > C. The sequencing peak map and KASPar genotyping map are shown in Figs. 2 and 3.

### Genotyping and allele frequency analysis

The primers in Table 2 were used for the KASPar genotyping of two SNPs. There are three genotypes of the *AGO2* gene, which were AA, CA and CC genotypes. Their genotype frequencies were 0.40, 0.35 and 0.25, respectively. Expected heterozygosity (*He*), expected homozygosity (*Ho*), allele number (*Ne*), polymorphism information content (*PIC*) and the *P* value of Hardy-Weinberg Equilibrium (*PHWE*) were calculated based on previous research^41^. *Ne*, *Ho*, *He* and *PIC* of the *AGO2* gene were 1.94, 0.51, 0.49 and 0.37, respectively. There are three genotypes of the *PLEC* gene, which were TT, TC and CC genotypes. Their genotype frequencies were 0.07, 0.38 and 0.55, respectively. *Ne*, *Ho*, *He* and *PIC* of *PLEC* gene were 1.62, 0.62, 0.38 and 0.31, respectively (Table 3).

**Table 2.** KASPar genotyping primer.

Gene name	Primer name	Primer sequence (5′–3′)
AGO2	Primer_AlleleX	GAAGGTGACCAAGTTCATGCTGCAGAACCACCTCTTGCAAATGAA
	Primer_AlleleY	GAAGGTCGGAGTCAACGGATTCAGAACCACCTCTTGCAAATGAC
	Primer_Common	CAAGGGAAGCCCCGTTTCAAAGG
PLEC	Primer_AlleleX	GAAGGTGACCAAGTTCATGCTAAGGTGTCCACTTGCTTGTTCC
	Primer_AlleleY	GAAGGTCGGAGTCAACGGATTGAAGGTGTCCACTTGCTTGTTCT
	Primer_Common	GACTCAGCCTGGAACTGACTGGT

**Table 3.** Genotype frequency, allele frequency and genetic diversity at the *AGO2* g.51700 a > C and *PLEC* g.23157 C > T loci.

Loci	No.	Genotype	Genotype frequency	Allele	Allele frequency	*He*	*Ho*	*Ne*	*PIC*	*PHWE*
*AGO2* g.51700 A > C	AA	505	0.4	A	0.57	1.94	0.51	0.49	0.37	0.58
	CA	485	0.35							
	CC	307	0.25	C	0.43					
*PLEC* g.23157 C > T	TT	89	0.07	T	0.26	1.62	0.62	0.38	0.31	0.26
	TC	500	0.38							
	CC	724	0.55	C	0.74					

*He*: Expected heterozygosity; *Ho*: expected homozygosity; *Ne*: effective allele number; *PIC*: polymorphism information content; *PHWE*: *P* value of the Hardy-Weinberg equilibrium.

### Association analysis of combined genotypes of AGO2 and PLEC genes with body size traits

In this study, body size traits (including body height, body length, chest circumference and cannon circumference) were measured in the sheep population at different periods. We used a general linear model to analyze the association between the SNPs of *AGO2* and *PLEC* genes and body size traits. The mutation at the *AGO2* g.51700A > C loci in sheep resulted in the CC genotype individuals’ chest circumference of 160 and 180 d were significantly higher than those of AA genotype individuals’ cannon circumference of 100, 120, 140, 160 and 180 day (*p* < .05). For *PLEC* gene, SNP of *PLEC* had significant differences with body height and body length. CC genotype individuals were significantly different from TT genotype individuals in body height at 160 and 180 days, length at 120, 140 and 180 days (*p* < .05) (Table 4).

**Table 4.** Association analysis of *AGO2* g.51700 a > C and *PLEC* g.23157 C > T with body size traits.

Items	*AGO2* g.51700A > C						*PLEC* g.23157 C > T					
	AA	CA	CC	*p*-value			TT	TC	CC	*p*-value		
No.	505	485	307	AA-CA	AA-CC	CA-CC	89	500	724	TT-TC	TT-CC	TC-CC
Body height 80	54.57 ± 0.15	54.63 ± 0.15	54.43 ± 0.19	.79	.57	.42	54.17 ± 0.36	54.60 ± 0.15	54.60 ± 0.12	.26	.26	.99
Body height 100	57.98 ± 0.16	57.96 ± 0.16	58.07 ± 0.20	.94	.74	.70	57.56 ± 0.38	58.06 ± 0.16	57.99 ± 0.13	.22	.28	.73
Body height 120	61.24 ± 0.15	61.03 ± 0.15	61.48 ± 0.19	.35	.32	.07	60.94 ± 0.36	61.19 ± 0.15	61.30 ± 0.12	.51	.34	.57
Body height 140	63.82 ± 0.16	63.69 ± 0.16	63.92 ± 0.20	.55	.71	.37	63.03 ± 0.37^b^	63.90 ± 0.16^a^	63.83 ± 0.13^ab^	.03	.04	.72
Body height 160	66.01 ± 0.16	65.88 ± 0.16	66.11 ± 0.20	.59	.70	.40	65.23 ± 0.38^b^	66.03 ± 0.16^ab^	66.04 ± 0.13^a^	.05	.05	.97
Body height 180	68.18 ± 0.17	68.36 ± 0.17	68.43 ± 0.21	.44	.34	.79	67.25 ± 0.40^b^	68.2 ± 0.17^ab^	68.49 ± 0.14^a^	.03	<.01	.18
Body length 80	56.02 ± 0.16	56.29 ± 0.16	56.06 ± 0.20	.25	.88	.40	55.53 ± 0.37	56.06 ± 0.16	56.23 ± 0.13	.19	.08	.40
Body length 100	60.95 ± 0.18	61.09 ± 0.18	61.35 ± 0.23	.58	.16	.37	60.50 ± 0.42	61.18 ± 0.18	61.09 ± 0.15	.14	.19	.71
Body length 120	65.21 ± 0.17	65.01 ± 0.18	65.41 ± 0.22	.41	.49	.16	64.57 ± 0.41^b^	64.92 ± 0.17^b^	65.49 ± 0.14^a^	.44	.04	.01
Body length 140	67.82 ± 0.16	67.62 ± 0.17	68.13 ± 0.21	.36	.25	.05	66.84 ± 0.39^b^	67.62 ± 0.17^b^	68.08 ± 0.14^a^	.07	<.01	.03
Body length 160	70.22 ± 0.16	70.27 ± 0.16	70.70 ± 0.20	.90	.07	.09	70.16 ± 0.38	70.25 ± 0.16	70.47 ± 0.13	.83	.45	.30
Body length 180	73.17 ± 0.19	73.21 ± 0.20	73.29 ± 0.25	.91	.70	.78	72.30 ± 0.47^b^	73.21 ± 0.20^ab^	73.31 ± 0.16^a^	.07	.04	.69
Chest circumference 80	60.97 ± 0.22	61.49 ± 0.22	61.01 ± 0.28	.11	.90	.20	60.95 ± 0.53	61.21 ± 0.22	61.20 ± 0.18	.66	.66	.97
Chest circumference 100	64.79 ± 0.23^b^	65.47 ± 0.23^a^	65.20 ± 0.29^ab^	.04	.28	.49	65.06 ± 0.55	65.21 ± 0.23	65.09 ± 0.19	.81	.96	.71
Chest circumference 120	69.41 ± 0.20	69.79 ± 0.21	69.85 ± 0.26	.22	.19	.81	69.56 ± 0.49	69.59 ± 0.21	69.72 ± 0.17	.95	.76	.63
Chest circumference 140	72.97 ± 0.22^b^	73.74 ± 0.22^a^	73.60 ± 0.28^ab^	.02	.08	.74	73.03 ± 0.52	73.40 ± 0.22	73.44 ± 0.18	.51	.46	.89
Chest circumference 160	77.17 ± 0.22^b^	77.80 ± 0.22^ab^	78.17 ± 0.28^a^	.05	.01	.28	77.60 ± 0.53	77.59 ± 0.22	77.67 ± 0.18	.98	.91	.78
Chest circumference 180	82.00 ± 0.22^b^	82.78 ± 0.22^a^	82.83 ± 0.28^a^	.02	.02	.82	81.98 ± 0.53	82.41 ± 0.22	82.58 ± 0.18	.45	.28	.56
Cannon circumference 80	6.80 ± 0.03	6.77 ± 0.03	6.75 ± 0.04	.47	.29	.68	6.76 ± 0.07	6.76 ± 0.03	6.80 ± 0.02	.98	.55	.26
Cannon circumference 100	7.10 ± 0.03^b^	7.18 ± 0.03^ab^	7.2 ± 0.040^a^	.06	.04	.63	7.14 ± 0.07	7.17 ± 0.03	7.14 ± 0.03	.71	.93	.57
Cannon circumference 120	7.38 ± 0.03^b^	7.46 ± 0.03^ab^	7.51 ± 0.04^a^	.05	.01	.28	7.47 ± 0.07	7.43 ± 0.03	7.44 ± 0.02	.60	.70	.77
Cannon circumference 140	7.67 ± 0.03^b^	7.72 ± 0.03^ab^	7.8 ± 0.04^a^	.18	<.01	.08	7.81 ± 0.07	7.69 ± 0.03	7.72 ± 0.02	.09	.17	.48
Cannon circumference 160	7.84 ± 0.03^b^	7.90 ± 0.03^b^	7.99 ± 0.04^a^	.20	<.01	.04	8.01 ± 0.07	7.89 ± 0.03	7.88 ± 0.02	.10	.08	.89
Cannon circumference 180	8.06 ± 0.03^b^	8.12 ± 0.03^b^	8.21 ± 0.04^a^	.15	<.01	.04	8.19 ± 0.07	8.11 ± 0.03	8.11 ± 0.02	.25	.25	.96

SNP: single nucleotide polymorphism. Values for the phenotypic data are shown as the mean ± standard error. Different superscript lowercase letters against values in the same column indicate significant differences (*p <* .05).

### Association analysis of combined genotypes of AGO2 and PLEC genes with body size traits

The association analysis of different genotypes random combinations of *AGO2* g.51700 A > C and *PLEC* g.23157 C > T on body height, body length, chest circumference, and cannon circumference of Hu sheep is shown in Table 5. The results showed that the body height and body length of sheep with AA*^AGO2^*-TT*^PLEC^* and CA*^AGO2^*-TC*^PLEC^* genotypes were significantly lower than those with AA*^AGO2^*-CC*^PLEC^*, CA*^AGO2^*-CC*^PLEC^*, CC*^AGO2^*-TC*^PLEC^* genotypes; The chest circumference and cannon circumference of individuals with AA*^AGO2^*-TT*^PLEC^* and AA *^AGO2^*-CC*^PLEC^* genotypes were significantly lower than those with CA*^AGO2^*-CC*^PLEC^* and CC*^AGO2^*-TC*^PLEC^* genotypes (*p* < 0.05). Therefore, the CA*^AGO2^*-CC*^PLEC^* and CC*^AGO2^*-TC*^PLEC^* genotypes are the dominant genotype combinations for improving body size traits in Hu sheep.

**Table 5.** The association analysis of combined genotypes at the *AGO2* and *PLEC* loci and Hu sheep body size traits.

Genotype									
Items	AA*^AGO2^*-TT*^PLEC^*	AA*^AGO2^*-TC*^PLEC^*	AA*^AGO2^*-CC*^PLEC^*	CA*^AGO2^*-TT*^PLEC^*	CA*^AGO2^*-TC*^PLEC^*	CA*^AGO2^*-CC*^PLEC^*	CC*^AGO2^*-TT*^PLEC^*	CC*^AGO2^*-TC*^PLEC^*	CC*^AGO2^*-CC*^PLEC^*
No.	40	191	267	35	186	256	13	110	178
Body height 80	53.81 ± 0.53	54.88 ± 0.24	54.50 ± 0.21	54.81 ± 0.57	54.34 ± 0.25	54.84 ± 0.21	53.54 ± 0.93	54.45 ± 0.32	54.46 ± 0.25
Body height 100	57.21 ± 0.56	58.32 ± 0.26	57.87 ± 0.22	58.01 ± 0.60	57.65 ± 0.26	58.18 ± 0.22	57.39 ± 0.99	58.20 ± 0.34	58.00 ± 0.27
Body height 120	60.64 ± 0.53^ab^	61.24 ± 0.24^ab^	61.35 ± 0.21^ab^	61.23 ± 0.57^ab^	60.75 ± 0.25^b^	61.26 ± 0.21^ab^	61.08 ± 0.94^ab^	61.73 ± 0.32^a^	61.32 ± 0.25^ab^
Body height 140	62.54 ± 0.55^b^	64.14 ± 0.25^a^	63.84 ± 0.21^a^	63.31 ± 0.59^ab^	63.39 ± 0.26^b^	63.98 ± 0.22^a^	63.77 ± 0.97^ab^	64.23 ± 0.33^a^	63.68 ± 0.26^ab^
Body height 160	64.76 ± 0.57^b^	66.19 ± 0.26^a^	66.12 ± 0.22^a^	65.73 ± 0.61^ab^	65.49 ± 0.26^b^	66.21 ± 0.22^a^	65.31 ± 0.99^ab^	66.61 ± 0.34^a^	65.84 ± 0.27^a^
Body height 180	66.86 ± 0.59^b^	68.21 ± 0.27^a^	68.40 ± 0.23^a^	67.53 ± 0.63^ab^	68.00 ± 0.27^b^	68.75 ± 0.23^a^	67.69 ± 1.03^ab^	68.48 ± 0.35^a^	68.39 ± 0.28^a^
Body length 80	55.26 ± 0.55	56.05 ± 0.25	56.11 ± 0.21	56.11 ± 0.59	56.14 ± 0.26	56.42 ± 0.22	54.81 ± 0.97	55.89 ± 0.33	56.20 ± 0.26
Body length 100	60.12 ± 0.63^b^	61.23 ± 0.29^ab^	60.83 ± 0.24^ab^	61.07 ± 0.67^ab^	60.80 ± 0.29^ab^	61.32 ± 0.25^ab^	60.15 ± 1.10^ab^	61.60 ± 0.38^a^	61.25 ± 0.30^ab^
Body length 120	64.31 ± 0.61	65.01 ± 0.28	65.52 ± 0.24	64.91 ± 0.66	64.52 ± 0.28	65.43 ± 0.24	64.46 ± 1.07	65.26 ± 0.37	65.54 ± 0.29
Body length 140	66.63 ± 0.59^b^	67.67 ± 0.27^ab^	68.13 ± 0.23^a^	66.96 ± 0.63^ab^	67.10 ± 0.27^b^	68.12 ± 0.23^a^	67.15 ± 1.03^ab^	68.31 ± 0.35^a^	68.07 ± 0.28^a^
Body length 160	69.81 ± 0.57^ab^	70.13 ± 0.26^ab^	70.37 ± 0.22^ab^	70.44 ± 0.61^ab^	69.87 ± 0.26^b^	70.56 ± 0.22^a^	70.50 ± 1.00^ab^	70.96 ± 0.34^a^	70.5 ± 0.27^ab^
Body length 180	71.62 ± 0.69^b^	73.29 ± 0.32^a^	73.32 ± 0.27^a^	72.80 ± 0.74^ab^	72.87 ± 0.32^ab^	73.53 ± 0.27^a^	73.08 ± 1.21^ab^	73.61 ± 0.42^a^	73.01 ± 0.33^ab^
Chest circumference 80	60.51 ± 0.78	61.12 ± 0.36	60.95 ± 0.30	61.59 ± 0.83	61.48 ± 0.36	61.58 ± 0.31	60.62 ± 1.37	60.86 ± 0.47	61.16 ± 0.37
Chest circumference 100	64.39 ± 0.82^ab^	64.97 ± 0.37^ab^	64.68 ± 0.32^b^	65.69 ± 0.87^ab^	65.31 ± 0.38^ab^	65.68 ± 0.32^a^	65.46 ± 1.43^ab^	65.35 ± 0.49^ab^	65.11 ± 0.39^ab^
Chest circumference 120	69.54 ± 0.73^ab^	69.67 ± 0.33^ab^	69.21 ± 0.28^b^	69.90 ± 0.78^ab^	69.26 ± 0.34^b^	70.22 ± 0.29^a^	68.73 ± 1.27^ab^	69.95 ± 0.44^ab^	69.92 ± 0.34^ab^
Chest circumference 140	72.05 ± 0.77^b^	73.21 ± 0.35^ab^	72.92 ± 0.30^b^	74.20 ± 0.82^ab^	73.33 ± 0.36^ab^	74.06 ± 0.31^a^	72.89 ± 1.35^ab^	73.85 ± 0.47^a^	73.52 ± 0.37^ab^
Chest circumference 160	76.48 ± 0.78^ab^	77.34 ± 0.36^ab^	77.13 ± 0.30^b^	78.61 ± 0.84^ab^	77.39 ± 0.36^ab^	78.04 ± 0.31^a^	78.35 ± 1.37^ab^	78.33 ± 0.47^a^	78.03 ± 0.37^ab^
Chest circumference 180	81.28 ± 0.78^b^	82.14 ± 0.36^b^	82.03 ± 0.30^b^	83.06 ± 0.84^ab^	82.66 ± 0.36^ab^	82.81 ± 0.31^ab^	81.23 ± 1.37^ab^	82.51 ± 0.47^ab^	83.18 ± 0.37^a^
Cannon circumference 80	6.71 ± 0.10	6.78 ± 0.05	6.83 ± 0.04	6.82 ± 0.11	6.76 ± 0.05	6.77 ± 0.04	6.73 ± 0.18	6.7 ± 0.06	6.78 ± 0.05
Cannon circumference 100	6.92 ± 0.11^b^	7.12 ± 0.05^ab^	7.11 ± 0.04^ab^	7.31 ± 0.11^a^	7.19 ± 0.05^a^	7.18 ± 0.04^a^	7.35 ± 0.19^a^	7.21 ± 0.06^a^	7.18 ± 0.05^a^
Cannon circumference 120	7.31 ± 0.10^b^	7.39 ± 0.05^b^	7.37 ± 0.04^b^	7.52 ± 0.11^ab^	7.43 ± 0.05^b^	7.48 ± 0.04^ab^	7.79 ± 0.18^a^	7.48 ± 0.06^ab^	7.51 ± 0.05^a^
Cannon circumference 140	7.61 ± 0.10^b^	7.64 ± 0.05^b^	7.69 ± 0.04^b^	7.94 ± 0.11^a^	7.69 ± 0.05^b^	7.72 ± 0.04^b^	8.09 ± 0.17^a^	7.81 ± 0.06^ab^	7.77 ± 0.05^ab^
Cannon circumference 160	7.82 ± 0.10^b^c	7.87 ± 0.05^b^c	7.82 ± 0.04c	8.11 ± 0.11^ab^	7.86 ± 0.05^b^c	7.90 ± 0.04^b^c	8.39 ± 0.18^a^	7.98 ± 0.06^b^	7.98 ± 0.05^b^
Cannon circumference 180	8.08 ± 0.10^ab^	8.05 ± 0.05^b^	8.06 ± 0.04^b^	8.23 ± 0.11^ab^	8.11 ± 0.05^ab^	8.11 ± 0.04^ab^	8.42 ± 0.17^a^	8.24 ± 0.06^a^	8.19 ± 0.05^a^

Values for the phenotypic data are shown as the mean ± standard error. Different superscript lowercase letters against values in the same column indicate significant differences (*P* < 0.05).

The AA*^AGO2^*-TC*^PLEC^*, AA*^AGO2^*-CC*^PLEC^*, CA*^AGO2^*-CC*^PLEC^* and CC*^AGO2^*-TC*^PLEC^* genotypes individuals were higher than that of AA*^AGO2^*-TT*^PLEC^* and CA*^AGO2^*-TC*^PLEC^* genotype individuals on body height at 140 and 180 days (*p* < .05), and CC*^AGO2^*-CC*^PLEC^* genotype individuals on body height was higher than AA*^AGO2^*-TT*^PLEC^* and CA*^AGO2^*-TC*^PLEC^* at 160 and 180 days. For body length, CC*^AGO2^*-TC*^PLEC^* genotype individuals was longer than AA*^AGO2^*-TT*^PLEC^* genotype individuals at 100 days, AA*^AGO2^*-CC*^PLEC^*, CA*^AGO2^*-CC*^PLEC^*, CC*^AGO2^*-TC*^PLEC^*, CC*^AGO2^*-TT*^PLEC^* genotypes individuals was longer than AA*^AGO2^*-TT*^PLEC^* and CA*^AGO2^*-TC*^PLEC^* genotype individuals at 140 days, CA*^AGO2^*-CC*^PLEC^* and CC*^AGO2^*-TC*^PLEC^* genotypes individuals were longer than CA*^AGO2^*-TC*^PLEC^* genotype individuals at 160 days and AA*^AGO2^*-TC*^PLEC^*, AA *^AGO2^*-CC*^PLEC^*, CA*^AGO2^*-CC*^PLEC^* and CC*^AGO2^*-TC*^PLEC^* genotypes individuals was longer than AA*^AGO2^*-TT*^PLEC^* genotype individuals at 180 d (*p* < .05). Furthermore, the CA*^AGO2^*-CC*^PLEC^* genotype individuals were significantly higher than that of AA*^AGO2^*-CC*^PLEC^* genotype individuals on chest circumference at 100, 120, 140, 160 days (*p* < .05). CC*^AGO2^*-TT*^PLEC^* genotype is the dominant genotype that promotes the development of cannon circumference in 100–180 d. In conclusion, it can be found that with the increase of C alleles at *AGO2* g.51700 A > C and *PLEC* g.23157 C > T, the body size traits of sheep gradually increased.

### Expression profile analysis

**Figure 4.**
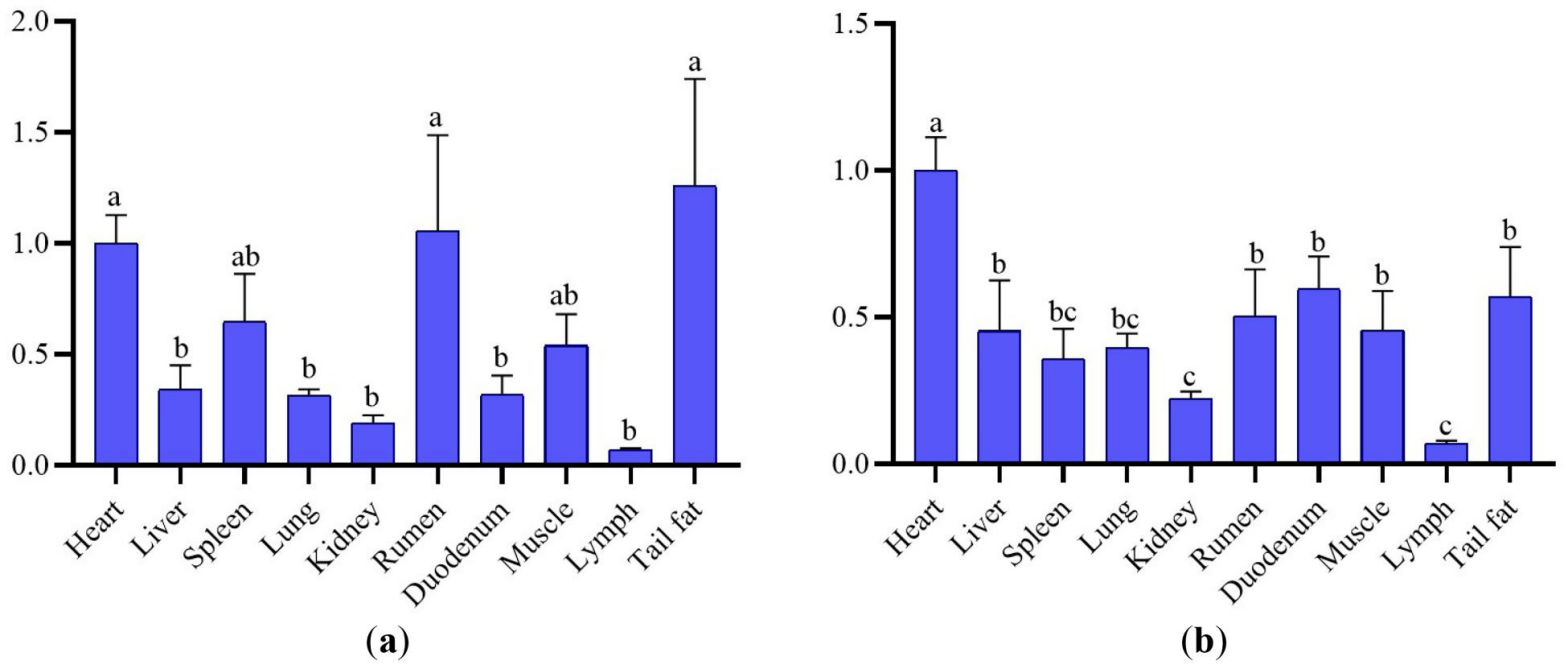
The mRNA expression level of *AGO2* (a) and *PLEC* (b) genes in different tissues of Hu sheep. Different lowercase letters indicate significant differences (*p <* .05).

Collect tissue samples from the heart, liver, spleen, lungs, kidneys, rumen, duodenum, muscles, lymph, and tail fat of six randomly tested Hu sheep for measuring the expression levels of *AGO2* and *PLEC* genes. Perform qRT-PCR according to the primer sequence in Table 6. The expression of the *AGO2* gene is shown in Fig. 4(a). It is the high expression in sheep heart, rumen and tail fat (*p* < .05), the low expression in the liver, lung, kidney, duodenum and lymph (*p* < .05). It is worth noting that the expression of the *AGO2* gene in liver and lymph of different sheep individuals is very different. Figure 4(b) shows the expression of the *PLEC* gene in Hu sheep. The expression of the *PLEC* gene was the highest in the heart and the lowest in lymph (*p* < .05). The expression of the *PLEC* gene in the heart, rumen and tail fat was slightly higher than that in spleen and muscle, and significantly higher than that in liver, lung, kidney, duodenum and lymph (*p* < .05).

**Table 6.** Forward and reverse primers, optimal annealing temperature and product length of RT-qPCR for the *AGO2* and *PLEC* genes.

Gene	Primer Name	Primer sequence (5′–3′)	Tm(°C)	Size(bp)
AGO2	AGO2-q-F	CAAGCCCATGCTCATCGACA	58.6	263
	AGO2-q-R	AGCACTAACTCCCGGTACTCC		
PLEC	PLEC-q-F	CGTCGGCAATGTCATCGTTC	58.8	245
	PLEC-q-R	CGCTCCATCACCGTCACAC		

## Discussion

Single-nucleotide polymorphisms (SNPs) are not only one of the most common forms of genetic variation in biology but also one of the reasons for the differences in quantitative traits of animals^42^. Mutations are divided into synonymous mutations and missense mutations. Missense mutations occur in the DNA coding region, causing changes in the types of amino acids. It is one of the most numerous mutations in protein-alteration^43^. The previous study suggested that about 2% of people carry missense mutations any given gene^44^. Missense mutations often cause phenotypic changes or increase the incidence of diseases^4^^,^^45^^,^^46^. Synonymous mutations are called silent mutations, do not alter the amino acid encoded by the affected codon due to the degeneracy of the genetic code^47^. However, synonymous mutations can change the stability of mRNA, splicing regulatory sites and miRNA binding sites^47^. This experiment was designed to investigate the impact of genetic SNPs on the body size traits of Hu sheep and further improve their growth performance.

In this study, *AGO2* and *PLEC* genes were used as candidate genes to analyze the effect of SNPs of these two genes on body size traits and the effect of their combined action on body size in the sheep population. A synonymous mutation in *AGO2* and *PLEC* genes were found. The g.51700 C > A mutation of the *AGO2* gene was significantly correlated with the chest circumference and cannon circumference of sheep, and the *PLEC* g.23157 T > C gene was significantly correlated with the height and length of sheep.

*AGO2* has retained the ability to cleave target mRNAs guided by small-interfering RNAs (siRNAs) in addition non-cleavage miRNA based gene silencing^48^. Therefore, it is are ubiquitous in various organs of animals, and this argument has also been confirmed in this experiment^8^^,^^49–52^. In previous studies, the *AGO2* gene was mostly expressed in areas with active energy metabolism, which is consistent with the results of this study. *AGO2* expression is high in cardiac myocytes that maintain the heart beating and rumen that secretes a lot of enzymes. Hepatic Argonaute 2 (AGO2) – mediated RNA silencing regulations both intrinsic energy production and consumption and emissions energy metabolism in the causes of obesity^12^, which is supported by the argument in mice^53^. In addition, due to the expression of certain miRNAs and mRNA cleavage function of *AGO2*, the expression of a large number of miRNAs in the liver is affected. It is worth noting that miR-802, miR-103/107, miR-130a and miR-148a have all been shown to affect glucose and lipid metabolism^54–56^. When *AGO2* is lacking, the pathways of energy metabolism are affected, including fatty acid biosynthesis, AMPK signaling, protein processing, insulin signaling. etc., thereby affecting growth and development. This confirms the positive effect of the *AGO2* gene on animal growth traits, and to some extent explains the mechanisms of *AGO2* g.51700 A > C mutation improving the body size traits of Hu sheep.

The *PLEC* gene encodes plectin, which is expressed in a wide variety of tissues and cell types, and it is particularly abundant in tissues subjected to great mechanical stress, such as stratified and simple epithelia, skeletal and heart muscle, and blood vessels^57^^,^^58^. For the high expression of the *PLEC* gene in the heart, the results obtained in this paper are consistent with the existing studies^6^. Plectin plays a role in maintaining the tissue integrity of skin, striated muscle and heart^59^. An Icelandic study showed that the missense mutation of *PLEC* in the heart of local people was related to atrial fibrillation^60^. In addition, there was another case of epidermolysis bullosa caused by a nonsense mutation in the coding region *PLEC* c.7159 G > T located in exon 31, the patient showed serious skin and muscle disorders^61^. Research has shown that *PLEC* can effectively promote C2C12 myoblast differentiation and promotion, and can inhibit their apoptosis. Moreover, *PLEC* can also regulate the expression of atropin-1 and muRF-1 genes, thereby inhibiting muscle atrophy. In addition, *PLEC* can bind to Disheveled-2 (Dvl-2) and forms a protein complex, and then activate the Wnt signaling pathway, thereby playing an important role in the development of skeletal muscle and improving the growth performance of Hu sheep through its impact on skeletal muscle development^62^. In conclusion, *PLEC* is closely related to the normal function of skeletal muscle, myocardium and skin. Under the joint action of two genes, the body size trait of Hu sheep has been improved to a certain extent.

## Conclusions

The body size trait is one of the important economic traits in Hu sheep, which is regulated by multiple genes. Molecular Genetic marker assisted selection will help to promote the improvement of Hu sheep germplasm. The study identified polymorphic loci of *AGO2* and *PLEC* genes in Hu sheep using Sanger sequencing and KASPar genotyping techniques. Through the size of body height, body length, chest circumference and cannon circumference of Hu sheep at different stages, it was found that *AGO2* g.51700 A > C and *PLEC* g.23157 C > T were related to the body size traits of Hu sheep. *AGO2* g.51700 A > C mainly affected the cannon circumference and chest circumference of Hu sheep, and *PLEC* g.23157 C > T affected the body height and body length. The combined action of the two genes can significantly improve the body size traits of Hu sheep. The expression of *AGO2* in the heart, rumen and tail was higher than that in other parts, and the expression of the *PLEC* gene was the highest in the heart. Through experiments, it can be proved that *AGO2* and *PLEC* genes may be used as candidate genes to optimize sheep production performance and improve economic benefits in production. However, further research is needed to explore the relationship between these two genes and the body size traits of Hu sheep in expanding the experimental population.

## References

[1] Li X, Yang J, Shen M, et al. Whole-genome resequencing of wild and domestic sheep identifies genes associated with morphological and agronomic traits. *Nat Commun*. 2020;11(1):2815. 32499537 10.1038/s41467-020-16485-1 PMC7272655

[2] Zhang D, Zhang X, Li F, et al. Whole-genome resequencing identified candidate genes associated with the number of ribs in Hu sheep. *Genomics.* 2021;113(4):2077–2084. 33965549 10.1016/j.ygeno.2021.05.004

[3] Höck J, Meister G. The Argonaute protein family. *Genome Biol*. 2008;9(2):210–210. 18304383 10.1186/gb-2008-9-2-210 PMC2374724

[4] Bossaerts L, Van de Craen EH, Cacace R, Asselbergh B, Van Broeckhoven C. Rare missense mutations in ABCA7 might increase Alzheimer’s disease risk by plasma membrane exclusion. *Acta Neuropathol Commun*. 2022;10(1):43. 35361255 10.1186/s40478-022-01346-3 PMC8973822

[5] Cardinale CJ, March ME, Lin X, et al. Regulation of Janus kinase 2 by an inflammatory bowel disease causal non-coding single nucleotide polymorphism. *J Crohns Colitis*. 2020;14(5):646–653. 32271392 10.1093/ecco-jcc/jjz213

[6] Castañón MJ, Walko G, Winter L, Wiche G. Plectin-intermediate filament partnership in skin, skeletal muscle, and peripheral nerve. *Histochem Cell Biol*. 2013;140(1):33–53. 23748243 10.1007/s00418-013-1102-0 PMC3695321

[7] Chahal J, Gebert LFR, Gan HH, et al. miR-122 and Ago interactions with the HCV genome alter the structure of the viral 5' terminus. *Nucleic Acids Res*. 2019;47(10):5307–5324. 30941417 10.1093/nar/gkz194 PMC6547439

[8] Chen X-J, Zhang C-J, Wang Y-H, Jin Z-B. Retinal degeneration caused by Ago2 disruption. *Invest Ophthalmol Vis Sci*. 2021;62(12):14. 10.1167/iovs.62.12.14 PMC8447045 34529004

[9] Zhang H, Wang Y, Dou J, et al. Acetylation of AGO2 promotes cancer progression by increasing oncogenic miR-19b biogenesis. *Oncogene.* 2019;38(9):1410–1431. 30305728 10.1038/s41388-018-0530-7 PMC6372475

[10] Beck EA, Healey HM, Small CM, et al. Advancing human disease research with fish evolutionary mutant models. *Trends Genet*. 2022;38(1):22–44. 34334238 10.1016/j.tig.2021.07.002 PMC8678158

[11] Lin S, Gregory RI. MicroRNA biogenesis pathways in cancer. *Nat Rev Cancer*. 2015;15(6):321–333. 25998712 10.1038/nrc3932 PMC4859809

[12] Zhang C, Seo J, Murakami K, et al. Hepatic Ago2-mediated RNA silencing controls energy metabolism linked to AMPK activation and obesity-associated pathophysiology. *Nat Commun*. 2018;9(1):3658. 30201950 10.1038/s41467-018-05870-6 PMC6131149

[13] Bhattacharjee J, Borra VJ, Salem ESB, et al. Hepatic Ago2 regulates PPARalpha for oxidative metabolism linked to glycemic control in obesity and post bariatric surgery. *Endocrinology.* 2021;162(4):bqab007. 33567453 10.1210/endocr/bqab007 PMC7875175

[14] Wiche G. Plectin-mediated intermediate filament functions: why isoforms matter. *Cells.* 2021;10(8):2154. 34440923 10.3390/cells10082154 PMC8391331

[15] Zrelski MM, Kustermann M, Winter L. Muscle-related plectinopathies. *Cells.* 2021;10(9):2480. 34572129 10.3390/cells10092480 PMC8466646

[16] Rice SJ, Tselepi M, Sorial AK, et al. Prioritization of PLEC and GRINA as osteoarthritis risk genes through the identification and characterization of novel methylation quantitative trait loci. *Arthritis Rheumatol*. 2019;71(8):1285–1296. 30730609 10.1002/art.40849 PMC6790675

[17] Winter L, Staszewska I, Mihailovska E, et al. Chemical chaperone ameliorates pathological protein aggregation in plectin-deficient muscle. *J Clin Invest*. 2014;124(3):1144–1157. 24487589 10.1172/JCI71919 PMC3934181

[18] Gundesli H, Talim B, Korkusuz P, et al. Mutation in exon 1f of PLEC, leading to disruption of plectin isoform 1f, causes autosomal-recessive limb-girdle muscular dystrophy. *Am J Hum Genet*. 2010;87(6):834–841. 21109228 10.1016/j.ajhg.2010.10.017 PMC2997373

[19] Konieczny P, Fuchs P, Reipert S, et al. Myofiber integrity depends on desmin network targeting to Z-disks and costameres via distinct plectin isoforms. *J Cell Biol*. 2008;181(4):667–681. 18490514 10.1083/jcb.200711058 PMC2386106

[20] Rezniczek GA, Konieczny P, Nikolic B, et al. Plectin 1f scaffolding at the sarcolemma of dystrophic (mdx) muscle fibers through multiple interactions with beta-dystroglycan. *J Cell Biol*. 2007;176(7):965–977. 17389230 10.1083/jcb.200604179 PMC2064082

[21] Natsuga K, Nishie W, Shinkuma S, et al. Plectin deficiency leads to both muscular dystrophy and pyloric atresia in epidermolysis bullosa simplex. *Hum Mutat*. 2010;31(10):E1687–E1698. 20665883 10.1002/humu.21330 PMC3023027

[22] Raza SHA, Khan R, Gui L, et al. Bioinformatics analysis and genetic polymorphisms in genomic region of the bovine SH2B2 gene and their associations with molecular breeding for body size traits in qinchuan beef cattle. *Biosci Rep*. 2020;40(3):BSR20192113. 32110807 10.1042/BSR20192113 PMC7069895

[23] Gupta PK, Langridge P, Mir RR. Marker-assisted wheat breeding: present status and future possibilities. *Mol Breed*. 2009;26(2):145–161.

[24] Kushanov FN, Turaev OS, Ernazarova DK, et al. Genetic diversity, QTL mapping, and marker-assisted selection technology in cotton (Gossypium spp.). *Front Plant Sci*. 2021;12:779386. 34975965 10.3389/fpls.2021.779386 PMC8716771

[25] Janaki Ramayya P, Vinukonda VP, Singh UM, et al. Marker-assisted forward and backcross breeding for improvement of elite Indian rice variety Naveen for multiple biotic and abiotic stress tolerance. *PLOS One.* 2021;16(9):e0256721. 34473798 10.1371/journal.pone.0256721 PMC8412243

[26] Zhang J, Song Q, Cregan PB, Jiang GL. Genome-wide association study, genomic prediction and marker-assisted selection for seed weight in soybean (Glycine max). *Theor Appl Genet*. 2016;129(1):117–130. 26518570 10.1007/s00122-015-2614-x PMC4703630

[27] Ijaz B, Zhao N, Kong J, Hua J. Fiber quality improvement in upland cotton (Gossypium hirsutum L.): quantitative trait loci mapping and marker assisted selection application. *Front Plant Sci*. 2019;10:1585. 31921240 10.3389/fpls.2019.01585 PMC6917639

[28] Sandhu KS, Shiv A, Kaur G, et al. Integrated approach in genomic selection to accelerate genetic gain in sugarcane. *Plants*. 2022;11(16):2139. 36015442 10.3390/plants11162139 PMC9412483

[29] Sonesson AK. Within-family marker-assisted selection for aquaculture species. *Genet Sel Evol*. 2007;39(3):301–317. 17433243 10.1186/1297-9686-39-3-301 PMC2682828

[30] Hu G, Do DN, Gray J, Miar Y. Selection for favorable health traits: a potential approach to cope with diseases in farm animals. *Animals*. 2020;10(9):1717. 32971980 10.3390/ani10091717 PMC7552752

[31] Fathoni A, Boonkum W, Chankitisakul V, Duangjinda M. An appropriate genetic approach for improving reproductive traits in crossbred Thai-Holstein cattle under heat stress conditions. *Vet Sci*. 2022;9(4):163. 35448661 10.3390/vetsci9040163 PMC9031002

[32] White SN, Knowles DP. Expanding possibilities for intervention against small ruminant lentiviruses through genetic marker-assisted selective breeding. *Viruses.* 2013;5(6):1466–1499. 23771240 10.3390/v5061466 PMC3717717

[33] Lee KP, Anthony NB, Orlowski SK, Rhoads DD. SNP-based breeding for broiler resistance to ascites and evaluation of correlated production traits. *Hereditas.* 2022;159(1):9. 35090566 10.1186/s41065-022-00228-x PMC8796538

[34] Xu Q, Zhao J, Guo Y, et al. A Single-Nucleotide Polymorphism in the Promoter of Porcine ARHGAP24 Gene Regulates Aggressive Behavior of Weaned Pigs After Mixing by Affecting the Binding of Transcription Factor p53. *Front Cell Dev Biol*. 2022;10:839583. 35433684 10.3389/fcell.2022.839583 PMC9010951

[35] Kim YC, Jeong MJ, Jeong BH. Regulatory Single Nucleotide Polymorphism of the Bovine IFITM3 Gene Induces Differential Transcriptional Capacities of Hanwoo and Holstein Cattle. *Genes (Basel)*. 2021;12(11):1662. 34828268 10.3390/genes12111662 PMC8619045

[36] Pruthviraj DR, Usha AP, Venkatachalapathy RT. Identification of a Novel Single Nucleotide Polymorphism in Porcine Beta-Defensin-1 Gene. *Asian-Australas J Anim Sci*. 2016;29(3):315–320. 26950860 10.5713/ajas.15.0638 PMC4811780

[37] Zhao L, Li F, Yuan L, et al. Expression of ovine CTNNA3 and CAP2 genes and their association with growth traits. *Gene.* 2022;807:145949. 34481004 10.1016/j.gene.2021.145949

[38] Liu X, Usman T, Wang Y, et al. Polymorphisms in Epigenetic and Meat Quality Related Genes in Fourteen Cattle Breeds and Association with Beef Quality and Carcass Traits. *Asian-Australas J Anim Sci*. 2015;28(4):467–475. 25656186 10.5713/ajas.13.0837 PMC4341095

[39] Liufu S, Lan Q, Liu X, et al. Transcriptome Analysis Reveals the Age-Related Developmental Dynamics Pattern of the Longissimus Dorsi Muscle in Ningxiang Pigs. *Genes (Basel)*. 2023;14(5):1050. 37239410 10.3390/genes14051050 PMC10218200

[40] Livak KJ, Schmittgen TD. Analysis of relative gene expression data using real-time quantitative PCR and the 2(-Delta Delta C(T)) method. *Methods*. 2001;25(4):402–408. 11846609 10.1006/meth.2001.1262

[41] Zhao H, Wu X, Cai H, et al. Genetic variants and effects on milk traits of the caprine paired-like homeodomain transcription factor 2 (PITX2) gene in dairy goats. *Gene.* 2013;532(2):203–210. 24076438 10.1016/j.gene.2013.09.062

[42] Vallejos-Vidal E, Reyes-Cerpa S, Rivas-Pardo JA, et al. Single-nucleotide polymorphisms (SNP) mining and their effect on the tridimensional protein structure prediction in a set of immunity-related expressed sequence tags (EST) in Atlantic Salmon (Salmo salar). *Front Genet*. 2019;10:1406. 32174954 10.3389/fgene.2019.01406 PMC7056891

[43] Miosge LA, Field MA, Sontani Y, et al. Comparison of predicted and actual consequences of missense mutations. *Proc Natl Acad Sci U S A*. 2015;112(37):E5189–E5198. 26269570 10.1073/pnas.1511585112 PMC4577149

[44] Andrews TD, Sjollema G, Goodnow CC. Understanding the immunological impact of the human mutation explosion. *Trends Immunol*. 2013;34(3):99–106. 23333204 10.1016/j.it.2012.12.001

[45] Tokheim C, Karchin R. CHASMplus reveals the scope of somatic missense mutations driving human cancers. *Cell Syst*. 2019;9(1):9.e8–23.e8. 31202631 10.1016/j.cels.2019.05.005 PMC6857794

[46] Razafinjatovo C, Bihr S, Mischo A, et al. Characterization of VHL missense mutations in sporadic clear cell renal cell carcinoma: hotspots, affected binding domains, functional impact on pVHL and therapeutic relevance. *BMC Cancer.* 2016;16(1):638. 27530247 10.1186/s12885-016-2688-0 PMC4987997

[47] Sharma Y, Miladi M, Dukare S, et al. A pan-cancer analysis of synonymous mutations. *Nat Commun*. 2019;10(1):2569. 31189880 10.1038/s41467-019-10489-2 PMC6562042

[48] Landthaler M, Gaidatzis D, Rothballer A, et al. Molecular characterization of human Argonaute-containing ribonucleoprotein complexes and their bound target mRNAs. *RNA*. 2008;14(12):2580–2596. 18978028 10.1261/rna.1351608 PMC2590962

[49] Li J-N, Sun H-L, Wang M-Y, Chen P-S. E-cadherin interacts with posttranslationally-modified AGO2 to enhance miRISC activity. *Front Cell Dev Biol*. 2021;9:671244. 34291046 10.3389/fcell.2021.671244 PMC8287304

[50] Shankar S, Tien JC-Y, Siebenaler RF, et al. An essential role for Argonaute 2 in EGFR-KRAS signaling in pancreatic cancer development. *Nat Commun*. 2020;11(1):2817. 32499547 10.1038/s41467-020-16309-2 PMC7272436

[51] Machado-Pereira M, Saraiva C, Bernardino L, Cristovao AC, Ferreira R. Argonaute-2 protects the neurovascular unit from damage caused by systemic inflammation. *J Neuroinflammation*. 2022;19(1):11. 34991639 10.1186/s12974-021-02324-7 PMC8740421

[52] Xu Q, Hou Y-x, Langlais P, et al. Expression of the cereblon binding protein argonaute 2 plays an important role for multiple myeloma cell growth and survival. *BMC Cancer.* 2016;16(1):297. 27142104 10.1186/s12885-016-2331-0 PMC4855823

[53] Khamzina L, Veilleux A, Bergeron S, Marette A. Increased activation of the mammalian target of rapamycin pathway in liver and skeletal muscle of obese rats: possible involvement in obesity-linked insulin resistance. *Endocrinology.* 2005;146(3):1473–1481. 15604215 10.1210/en.2004-0921

[54] Kornfeld J-W, Baitzel C, Könner AC, et al. Obesity-induced overexpression of miR-802 impairs glucose metabolism through silencing of Hnf1b. *Nature.* 2013;494(7435):111–115. 23389544 10.1038/nature11793

[55] Lu L, Wang J, Lu H, et al. MicroRNA-130a and -130b enhance activation of hepatic stellate cells by suppressing PPARγ expression: A rat fibrosis model study. *Biochem Biophys Res Commun*. 2015;465(3):387–393. 26255201 10.1016/j.bbrc.2015.08.012

[56] Huang J-Y, Chou S-F, Lee J-W, et al. MicroRNA-130a can inhibit hepatitis B virus replication via targeting PGC1α and PPARγ. *RNA*. 2015;21(3):385–400. 25595716 10.1261/rna.048744.114 PMC4338335

[57] Wiche G, Krepler R, Artlieb U, Pytela R, Denk H. Occurrence and immunolocalization of plectin in tissues. *J Cell Biol*. 1983;97(3):887–901. 6350322 10.1083/jcb.97.3.887 PMC2112553

[58] Wiche G, Krepler R, Artlieb U, Pytela R, Aberer W. Identification of plectin in different human cell types and immunolocalization at epithelial basal cell surface membranes. *Exp Cell Res*. 1984;155(1):43–49. 6386498 10.1016/0014-4827(84)90766-3

[59] Wiche G. Role of plectin in cytoskeleton organization and dynamics. *J Cell Sci*. 1998;111(17):2477–2486. 9701547 10.1242/jcs.111.17.2477

[60] Thorolfsdottir RB, Sveinbjornsson G, Sulem P, et al. A missense variant in PLEC increases risk of atrial fibrillation. *J Am Coll Cardiol*. 2017;70(17):2157–2168. 29050564 10.1016/j.jacc.2017.09.005 PMC5704994

[61] Argyropoulou Z, Liu L, Ozoemena L, et al. A novel PLEC nonsense homozygous mutation (c.7159G > T; p.Glu2387*) causes epidermolysis bullosa simplex with muscular dystrophy and diffuse alopecia: a case report. *BMC Dermatol*. 2018;18(1):1–1. 29352809 10.1186/s12895-018-0069-x PMC5775598

[62] Yin H, Han S, Cui C, et al. Plectin regulates Wnt signaling mediated-skeletal muscle development by interacting with Dishevelled-2 and antagonizing autophagy. *Gene.* 2021;783:145562. 33705811 10.1016/j.gene.2021.145562

